# Herpesviruses and MicroRNAs: New Pathogenesis Factors in Oral Infection and Disease?

**DOI:** 10.3389/fimmu.2018.02099

**Published:** 2018-09-27

**Authors:** Afsar R. Naqvi, Jennifer Shango, Alexandra Seal, Deepak Shukla, Salvador Nares

**Affiliations:** ^1^Mucosal Immunology Lab, College of Dentistry, University of Illinois at Chicago, Chicago, IL, United States; ^2^Department of Microbiology and Immunology, University of Illinois at Chicago, Chicago, IL, United States; ^3^Department of Ophthalmology and Visual Sciences, University of Illinois Medical Center, Chicago, IL, United States

**Keywords:** herpesviruses, MicroRNAs, viral microRNA, periodontitis, oral inflammation, pulpitis, periimplantitis

## Abstract

The oral cavity incessantly encounters a plethora of microorganisms. Effective and efficient oral innate and adaptive immune responses are incumbent to maintain healthy mucosa. A higher prevalence of Human Herpesviruses (HHV), a family of large enveloped DNA viruses, has been reported in multiple oral inflammatory diseases suggesting their involvement in disease progression. However, the viral components contributing to oral disease remain obscure. MicroRNAs (miRNA) are non-protein coding, single stranded ribonucleic acid (RNA) molecules that post-transcriptionally regulate diverse messenger RNAs. Thus, miRNAs can control large repertoire of biological processes. Changes in miRNA expression are associated with various oral infections and diseases. Cellular miRNAs can act as pro- or anti-viral factors and dysregulation of host miRNA expression occurs during herpesviruses infection. This strongly suggest a critical role of cellular miRNAs in host-herpesvirus interaction. Interestingly, HHV also encode multiple miRNAs (called viral miRNAs) that may play key role in host-pathogen interaction by modulating both host biological pathways and controlling viral life cycle. Recent studies from our laboratory have identified viral miRNAs (v-miRs) in diseased oral tissue biopsies and demonstrate their immunomodulatory roles. This review discusses the association of miRNAs (both host and viral) and herpesviruses in the pathogenesis of oral inflammatory diseases.

## Introduction

The oral cavity, besides being a niche for diverse commensal microbes, is a gateway to the host for a plethora of microbes and antigens. Evidently, oral tissue homoestasis is under stringent control of mulitple endogenous and exogenous factors that work in synchorny to maintain a healthy microenvironment (1). Aberrant cellular, pathogenic and environmental cues may shift this equilibrium toward disease development or progresion (2, 3). Identification of novel etiological factors that regulate biological pathways related to disease are of paramount importance to our understanding of the pathobiology of infectious oral diseases. Employing such molecules for diagnostic or therapeutic purpose can faciliatate the development of new treatment modalities of oral infections.

The family of herpesviruses consists of over 100 members that infect a diverse group of organisms. Herpesviruses share a common structural organization that includes a 100–250 kb large linear double-stranded DNA genome packaged in an icosahedral capsid, which is surrounded by a layer of tegument proteins that is in turn, enclosed within a lipid envelope (4, 5). The viruses replicate in the nucleus and utilize cytoplasmic organelles for protein production and maturation (6, 7). All herpesviruses cause lifelong latent infections, which is marked by limited viral gene expression. As a result there is little or no obvious disease manifestations associated with latent infections, but latent viral genomes may reactivate causing new rounds of active virus replication and host morbidities (8). Human infections are caused by nine markedly different herpesviruses, which are classified into three subfamilies. The alphaherpesvirus family includes herpes simplex types 1 and 2 (HSV-1, HSV-2), and varicella-zoster virus (VZV). These are cytolytic viruses that infect a variety of human cell-types and establish asymptomatic latent infections in neurons of the peripheral nervous system. The betaherpesviruses, which show more selectivity for human cell types, include cytomegalovirus (CMV), HHV-6, and HHV-7. Members of the gammaherpesvirus subfamily are lymphotropic and include Epstein Barr Virus (EBV) and HHV-8. Both HHV-8 and EBV are considered important cofactors in oral and non-oral forms of malignancy.

Almost all human herpesviruses have been found to encode miRNAs. The number of miRNAs, however, varies tremendously among the human herpesviruses, with Varicella Zoster Virus (VZV) encoding no miRNAs to EBV with 44 known mature miRNAs. Numerous studies demonstrate that viral miRNAs strongly enhance viral pathogenesis, critically regulate herpesvirus life cycle switch, immune subversion, promote the establishment of a reservoir of latently infected cells (9–11). Supporting this, multiple clinical studies reported that accumulation of herpesviral miRNAs correlate with disease pathogenesis indicating their pathogenic role in infection and disease (12–16). The contribution of viral miRNAs in oral inflammatory diseases, however, remains understudied.

MicroRNAs (miRNAs) are small, non-coding RNAs of ~18–25 nucleotides that have gained extensive attention as critical regulators in complex gene networks (17, 18). MiRNAs function to produce two main outcomes: translational silencing or destabilization of mRNA. Translational silencing results in complete inhibition of protein production whereas destabilization of mRNA results in reduced amounts of available protein. MiRNAs typically regulate post-transcriptionally, when mRNAs are translated to protein and bind to the 3′ untranslated regions (UTR) of mRNA impacting transport of mRNA, translational efficiency, subcellular localization, and stability (17–19). The binding of miRNA to the 3′UTR of mRNA is complementary/sequence based. Perfect complementarity at the “seed sequence” (positions 2–7 from the 5′ end of the miRNA) is required, but otherwise the binding does not need to be completely complementary. Furthermore, it has been shown that a single miRNA can simultanouesly bind to multiple mRNAs. Therefore, the expression of a large number of genes may be regulated in parallel (20). By fine-tuning the transcriptome, miRNAs regulate key cellular processes including differentiation, apoptosis, immune cell lineage commitment, maturation, and maintenance of immune function (21–26). Recent reports indicate that pathogens modulate host miRNA expression patterns (27, 28) while changes in host miRNA profiles have been recognized in numerous diseases, including cancers, autoimmune, and inflammatory diseases (29–32).

The detection of viruses, predominantly of herpes family in oral tissues, was initially considered as mere tropism for cells of oral tissues. However, accumulating evidences of herpesvirus detection in different oral tissues and their higher prevalence in diseased tissues indicate a strong association with disease progression. In this review, we discuss the herpesviruses that predominantly cause oral infections and relate cellular and viral miRNAs as new etiological factors in the development of oral inflammtory diseases. Herpesviruses and miRNAs are widely acknowledged for their role in oral cancer development and metastasis, cancer prognosis and diagnosis and patient survival outcomes. However, these topics will not be covered in the current review. Several interesting articles have thoroughly covered the association of herpesvirus and miRNAs in oral cancer [reviewed by (33–37)]. The altered expression of cellular miRNAs has been reported by multiple groups incuding our laboratory. These changes in miRNA expression is proposed to modulate immune reponses, wound repair and tissue regeneration leading to disease manifestation. Here we also discuss contemporary understanding of herpesviruses and microRNA in the pathogenesis of oral inflammatory disesases and their role in the etiology of mucosal inflammatory diseases.

## Herpesviruses and oral inflammatory disease

**Figure 1:**
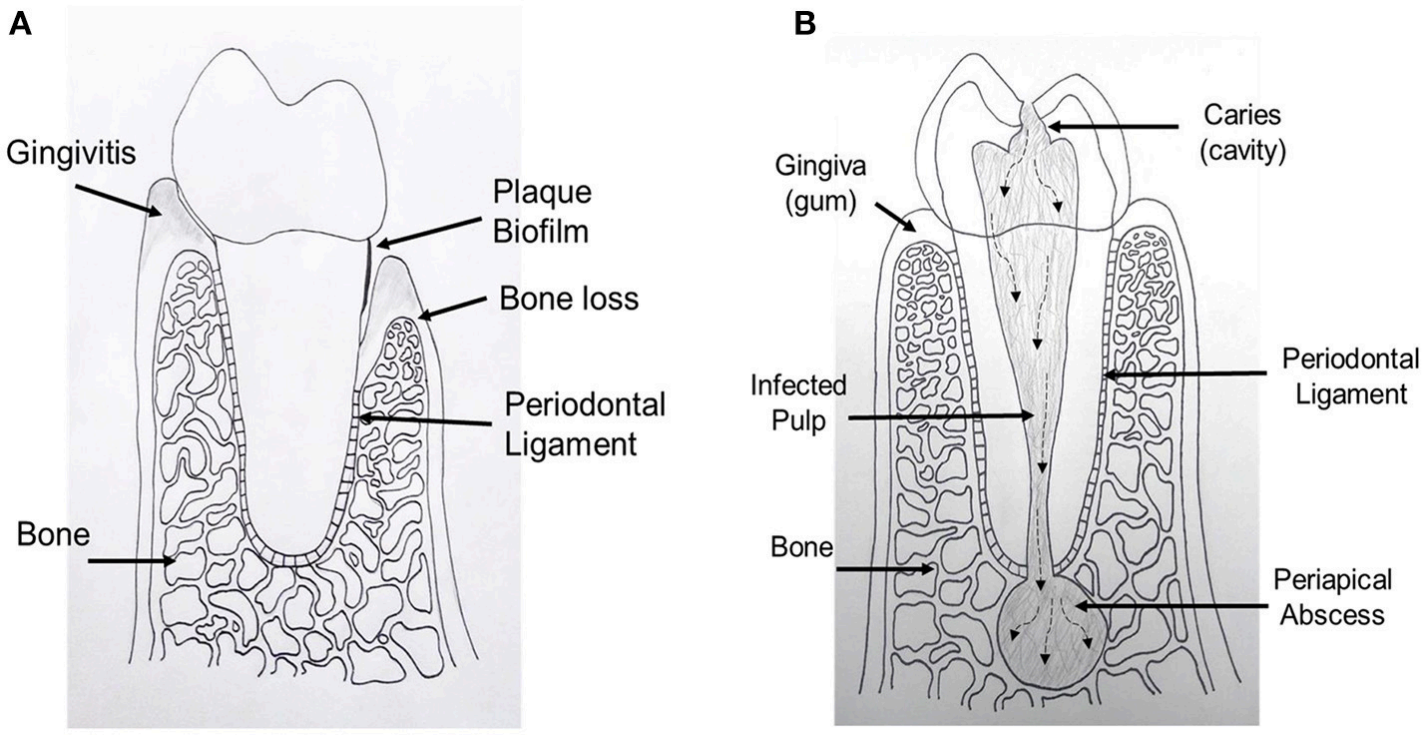
Schematic drawing shows periodontal and endodontic tissues. **(A)** Cross sectional diagram of alveolar bone surrounding a periodontally involved tooth. Left: gingivitis, gingival inflammation without alveolar bone loss. Right: periodontitis, alveolar bone loss and gingival tissue recession. **(B)** Cross sectional diagram of an endodontically involved tooth. A carious (cavity) lesion penetrates tooth structure and reaches the central pulp tissue. Over time, bacteria and their by-products move toward the end of the tooth and exit into the bone causing a periapical abscess.

Traditionally, the etiology of periodontal and periapical diseases were thought to be bacterial in nature. However, other etiologic factors such as viruses may contribute to the pathogenesis of periodontal and periapical diseases through multiple avenues. Viruses have the ability to damage periodontal or periapical tissues via lysis of host cells, facilitation of immune-mediated host destruction, or suppression of the immune system, making the host less able to resist bacterial challenge (38). As early as the 1960s, viruses had been identified in subacute and chronic periapical granulomas, but their exact role was unknown (39). Parra and Slots detected viruses from periodontal pockets using polymerase chain reaction (PCR) and later advocated herpesvirus as a cofactor in periodontal disease (38). Subsequently, Sabeti et al. described a similar relationship between concurrent herpesvirus infection and apical periodontitis to that of herpes and periodontal disease (40). Currently, herpesviruses have been isolated in various parts of the oral cavity and are increasingly acknowledged as potential etiological agent in the pathogenesis of oral diseases. Table 1 enlists summary of studies that examined the presence of herpesvirus derived nucleic acids (DNA or RNA) in major oral inflammatory disorders *viz*., periapical periodontitis, pulpitis, endodontic abscess, periodontitis and peri-implantitis. The definition of oral sites and diseases mentioned in the text are listed in Box 1 and a schematic description is provided in Figure 1.

**Table 1:** Summary of the selected observational studies that examined Herpesvirus prevalence in periodontal, endodontic, and peri-implantitis biopsies.

**Tissue biopsy disposition (subject sample size)**	**Herpesviruses investigated**	**Assays performed**	**Associations described**	**References**
*Periodontitis* Diseased (*n* = 14) Control (*n =* 11)	EBV (1 and 2), HCMV, HSV, HHV-6B, HHV-7, and KSHV	Viral DNA (PCR)	EBV1 (Disease-79%; Healthy-27%) EBV2 (Disease-50%; Healthy-0%) HCMV (Disease-86%; Healthy-18%) HSV (Disease-57%; Healthy-9%) HHV6B (Disease-21%; Healthy-0%) HHV-7 (Disease-43%; Healthy-0%) KSHV (Disease-29%; Healthy-0%)	(41)
*Periodontitis* Diseased (*n =* 20 CP and 10 AP) Control (*n =* 22)	HCMV	Viral DNA (PCR)	HCMV (53.3%)	(42)
*Periodontitis* Diseased (*n =* 24) Control (*n =* 13)	EBV, HCMV and HHV-7	Viral DNA and RNA (PCR)	EBV (Disease-50%; Healthy-7.7%) HHV-7 DNA (Disease-91.7%; Healthy-61.5%) HHV-7 RNA (Disease-15.4%; Healthy-15.4%) HCMV (Disease-4.1%; Healthy-0%)	(43)
*Periodontitis* Diseased (*n =* 20 each CP and AP) Healthy (*n =* 20)	EBV and HCMV	Viral DNA (PCR)	EBV-1 (AP-45%; CP-25%; Healthy-10%) HCMV (AP-45%; CP-20%; Healthy- 0%)	(44)
*Periapical lesions* (*n =* 5 symptomatic)	EBV, HCMV, and HSV-1	Viral RNA (PCR)	EBV (100%) HCMV (100%) HSV-1 (0%)	(40)
*Periapical lesion*	EBV, HCMV, and HSV-1	Viral RNA (PCR)	HCMV (79.4%) EBV (61.7%) HSV-1 (5.8%)	(45)
*Apical periodontitis* Diseased (*n =* 35: 26 HIV seronegative and 9 HIV-seropostive)	EBV and HCMV	Immunohisto-chemistry (Anti-HCMV M0854; Anti EBV LMP M0897)	EBV (31%) HCMV (23%) EBV+HCMV (14%)	(46)
*Apical periodontitis* Diseased (*n =* 17 symptomatic and *n =* 23 asymptomatic) Control (*n =* 40 healthy pulp)	EBV and HCMV	Viral DNA and RNA (PCR)	Diseased: EBV DNA (72.5%) and EBNA-2 RNA (50%) Control: EBV DNA and RNA (both 2.5%) Diseased: HCMV DNA and RNA (10%) Control: Not detected	(47)
*Apical periodontitis* Diseased (*n =* 12 symptomatic and *n =* 16 asymptomatic)	EBV and HCMV	Viral DNA (PCR)	HCMV Symptomatic (62.5%) HCMV Asymptomatic (41.7%) EBV Symptomatic (43.7 %) EBV Asymptomatic (25%)	(48)
*Periapical periodontitis* Diseased (*n =* 33: 20 symptomatic and 13 asymptomatic) Saliva (*n =* 15)	EBV and HCMV	Viral DNA (PCR)	EBV Symptomatic (70%) EBV Asymptomatic (38.5%) HCMV Symptomatic (15%) HCMV Asymptomatic (0%) EBV Saliva (40%) HCMV Saliva (6.7%)	(49)
*Apical abscess* Diseased (*n =* 33)	EBV (1&2), HCMV, HSV, HHV-6B, HHV-7, and KSHV	Viral DNA (PCR)	KSHV (54%); HHV-6 (6%); EBV (6%); VZV (6%)	(50)
*Endodontic pathoses* Diseased (*n =* 82 Pulp and periapical tissue) Control (*n =* 19 healthy pulp)	EBV, HCMV, HSV-1 and VZV	Viral DNA and RNA (PCR)	Diseased: EBV DNA (43.9%) and EBNA-2 RNA (25.6%) Control: EBV DNA and RNA (both 0%) Diseased: HCMV DNA (15.9%) and RNA (29.3%) Control: HCMV DNA (42.1%) and RNA (10.5%) Diseased: HSV-1 DNA (13.4%) and RNA (0%) Control: HCMV DNA (5.2%) and RNA (0%) Diseased: VZV DNA (3.4%) and RNA (0%) Control: HCMV DNA/RNA (both 0%)	(51)
*Peri-implantitis* Diseased (*n =* 30 peri-implantitis, *n =* 25 mucositis) Control (*n =* 25 healthy peri-implant sites)	EBV and HCMV	Viral DNA (PCR)	Diseased: EBV DNA (46.6%) Control: EBV DNA (3.3%) Diseased: HCMV DNA (53.3%) Control: HCMV DNA not detected	(52)
*Peri-implantitis* Diseased (*n =* 40) Control (*n =* 40)	HSV-1	Viral DNA (PCR)	Diseased (33.3) Control (28.3%)	(53)
*Peri-implantitis* Diseased (*n =* 23) Control (*n =* 23 contralateral)	EBV and HCMV	Viral DNA (PCR)	Diseased: EBV DNA (39%) Control: EBV DNA (4.3%) Diseased: HCMV DNA (4.3%) Control: HCMV DNA not detected	(54)

*AP, Aggressive Periodontitis; CP, Chronic Periodontitis*.

Box 1 **Definition of oral sites and diseases.**

1. **Periodontitis or Periodontal Disease:** Commonly referred to as “gum disease” is a group of infectious, inflammatory conditions affecting the supporting structures of teeth. The mildest form of periodontal disease is gingivitis, a dental biofilm-induced disease manifested as red, swollen, and bleeding gingiva (gums). Gingivitis does not induce loss of supporting bone and is reversible with treatment. Without treatment, the biofilm can advance below the gum line triggering a more severe form of inflammation resulting in loss of supporting alveolar bone surrounding teeth. The two major forms of periodontitis are chronic periodontitis and aggressive periodontitis.
2. **Chronic Periodontitis:** Chronic periodontitis is the most frequent form of periodontitis observed as a slow and progressive loss of tooth attachment. The inflammation triggers pocket formation and/or gum recession and loss of bone supporting the teeth. If untreated, it can result in tooth loss.
3. **Aggressive Periodontitis:** Aggressive periodontitis a rapidly progressive and aggressive form of periodontitis often observed in younger patients. Familial aggregation is often observed.
4. **Peri-Implantitis:** A progressive and irreversible disease of dental implant-surrounding bone and gingival tissues and is accompanied by increased pocket formation with bone resorption, decreased osseointegration, and purulence. If untreated, may lead to loss of the dental implant.
5. **Apical Periodontitis:** Inflammation and destruction of periradicular tissues of the tooth that occurs as a result of microbial, mechanical or chemical insults to the tooth dental pulp. The immune response leads to the formation of a bony lesion due to toxic irritation from the infected root canal system. The disease could be asymptomatic or symptomatic.
6. **Apical Abscess:** An acute inflammatory reaction to tooth pulpal infection and necrosis resulting in a rapid-onset of spontaneous pain, tenderness, purulence formation, and swelling of local tissues.

### Herpesviruses in endodontal tissues

Related to tooth pulpal inflammation, studies have investigated the presence of viruses and their possible role in pathology. Different HHV have been examined and detected in periapical periodontitis, apical abscess, and pulpitis strongly suggesting that endodontic tissues are a predominant niche for HHV. Specifically, HCMV, EBV, and KSHV exhibit propensity toward endodontic sites. Multiple studies have shown a viral presence in granulomas of symptomatic as well as asymptomatic periapical lesions. Although most of the studies used PCR based detection methods (40, 47, 48, 51), one particular study showed HHV presence using immunohistochemical staining against viral antigens (46). In two different cohorts of periapical lesions, Sabeti et al., detected viral transcripts of three herpesviruses (EBV, HCMV, and HSV-1) (40, 46). They observed higher prevalence of EBV and HCMV but very low detection rate for HSV-1 indicating that EBV and HCMV but not HSV-1 active infection is associated with periapical disease. Periapical lesions can be classified as symptomatic or asymptomatic. Three different groups investigated the presence of EBV and HCMV DNA or RNA in a large cohort of subjects with symptomatic or asymptomatic apical periodontitis and healthy tissues (45, 47, 49). These studies confirmed the detection of EBV and HCMV in diseased tissues but were rarely present in healthy tissues, based on the amplification of target HHV sites. Another common finding of these studies is that symptomatic periapical lesions tend to show higher HHV detection levels suggesting a probable association of HHV in the pathogenesis of these lesions. In a few studies, none of the prevalent HHV members was detected suggesting a probable influence of geographical location, disease stage, and biopsy collection sites. For instance, Heling et al., checked for HSV in pulpal or periapical lesions, while Roslaine et al., measured HSV, EBV, and HCMV genomes in pulpal tissues diagnosed with irreversible pulpitis (55, 56). None of these groups was able to detect HHV in pulp or periapical lesions by PCR. However, IgG/IgM antibodies against HSV were observed in the sera of 84% of the subjects indicating exposure to HSV (55).

Detection of different HHV was also reported in diseased pulp tissues. Ferriera et al. checked seven different HHV including EBV (1 and 2), HCMV, HSV, HHV-6B, HHV-7, and KSHV (50). More than half of the subjects were positive for KSHV genome, while HHV-6B, HHV-7, and VZV were detected in 6% of the biopsies. Similarly, diseased pulp and periapical tissues along with healthy pulps were evaluated for EBV, HCMV, HSV-1, and VZV genome and transcripts (51). In general, higher HHV DNA and RNA detection rate was observed in diseased compared with healthy tissues, except for HCMV where control tissues showed higher DNA detection than diseased tissues. PCR based methods showed the presence of multiple HHV in the same oral tissue sample indicating a possible coexistence of these viruses in infected cells. Indeed, using immunohistochemical staining Saboia-Dantas et al., showed co-localization of EBV and HCMV in apical periodontal tissues (46). These findings strongly confirmed that HHV could infect cells in close proximity. How infection with multiple HHV could exacerbate endodontic pathoses and the characterization of the underlying mechanisms of coinfection will require further studies.

Herpesviral-bacterial synergism might play a role in oral disease pathogenesis. To study this possible association, Verdugo et al., evaluated a correlation between HHV and periodontopathogens in periapical periodontitis (49). The pilot study collected symptomatic and asymptomatic periapical periodontitis lesions and salivary samples. They analyzed the presence of bacterial and viral DNA using PCR in granulation tissue derived from periapical periodontitis lesions. They found that symptomatic periapical periodontitis lesions had higher proportions of periodontopathogens and were 3.7 times more likely to be infected with EBV than asymptomatic periodontitis lesions. The periodontal pathogens *Treponema denticola, Prevotella intermedia, Aggregatibacter actinomycetemcomitans*, and *Porphyromonas gingivalis* were exhibited 1.6 times more in the symptomatic periapical periodontitis lesions as well. It was suggested that a causal relationship existed between EBV, specific bacterial anaerobic infection, and symptomatic periapical pathosis. Herpesvirus can contribute to periapical pathosis by causing uncontrolled release of tissue destructive cytokines, upregulate the growth of pathogenic bacteria, and initiate cytotoxic or immunopathologic events (57, 58). In a systematic review evaluating these two viruses, Slots et al., reported that herpesviruses are very common in symptomatic and large size periapical lesions, but that their role in pathogenesis is unclear (57). The detection of viral DNA and RNA did not relate specifically to the clinical features of apical periodontitis (59). Further studies aimed at gaining correlation between the viral genome detection, virus life cycle, clinical features of disease in larger cohort from different geographical locations can shed light on the relationship between peri-apical infections and periodontal pathogens.

### Herpesviruses in periodontal tissues

Herpesviruses have been studied extensively in periodontitis as well. Studies from the mid-1990s identified the high prevalence and high counts of HCMV, EBV, and HSV-1 in progressive periodontal disease [reviewed by (57, 60)]. Contreras et al., screened six predominant HHV members including EBV, HCMV, HSV, HHV-6B, HHV-7, and KSHV in diseased and control periodontal biopsies (41). Higher prevalence for all the viruses was observed in diseased compared with healthy tissues. Depending on the virus, the detection rate varied from approximately 30% (for KSHV) to 85% (EBV1) (41, 44, 61, 62). Some of these viruses (EBV1, HCMV, and HSV) were detected in healthy tissues, while others (EBV2, HHV-6B, HHV-7, and KSHV) were not detected in any of the control tissues (*n* = 11). Similarly, increased presence of EBV (50 vs. 7.7%), and HHV-7 (91 vs. 61.5%) but not HCMV (4.1 vs. 0%) was noticed in diseased and healthy periodontal tissues (43). However, Botero et al., showed increased detection of HCMV in chronic and aggressive periodontitis (42). These findings further suggest a strong association of HHV in the pathophysiology of oral diseases but also reinforce the notion that HHV detection varies with geographical location and disease state.

Based on the review of the literature on herpesviruses, Slots identified 26 recent studies from 15 countries on the prevalence of herpesviruses in periodontal tissues (60). He created median averages for all the studies on the prevalence of HCMV, EBV, and HSV-1 in aggressive and chronic periodontitis and compared with healthy periodontal tissues [Table 2; (60)]. The limitations of most of the studies related to the classification of disease activity in the periodontitis samples. Unfortunately, the population studies did not have clear criteria regarding the active disease status related to periodontitis. Nevertheless, these studies highlight the high detection rates of members of the *Herpesviridae* family in diseased periodontal tissues.

**Table 2:** Median percentages on the prevalence of HCMV, EBV, and HSV [Slots, (60)].

**Virus**	**Aggressive periodontitis (%)**	**Chronic periodontitis (%)**	**Healthy periodontium (%)**
HCMV	49	40	3
EBV	45	32	7
HSV	63	45	12

In relation to gingival disease, herpesviruses have been isolated in chronic gingivitis sites. The amounts however were quite low and were not believed to initiate gingivitis. The only studies that have shown higher amounts of EBV related to gingivitis are in studies of women with pregnancy gingivitis. It was reported that 39% of gingivitis sites and 40% of healthy periodontal sites harbored EBV (63).

### Herpesvirus in peri-implantitis

Lastly, herpesviruses have been studied in peri-implant lesions. The identification of EBV was seen in 39% of peri-implantitis lesions and in 4% of healthy peri-implant sites (54). Another study detected HCMV and EBV, respectively in 65 and 45% of peri-implantitis sites, whereas these viruses were detected in 22 and 33% of mucositis sites, and in 6 and 11% of healthy implant sites (52). The authors concluded that peri-implantitis and periodontitis exhibit comparable types and levels of herpesviruses infection. However, Parthiban et al., observed no significant differences in detection of HSV-1 in diseased and healthy peri-implant sites (53).

The direct relationship between the presence of herpesviruses and the unique disease manifestations during peri-apical, periodontal, and peri-implant pathology is unknown. The pathogenicity of herpesviruses is likely related to immune pathways and viral replication. Therefore targeting herpesviruses during periodontal therapy might transform current approaches for the diagnosis, prevention or treatment the disease. Although studies in this field are scarce, one group used this strategy to evaluate the HHV-periodontitis association. In this case report, the group employed antiviral drug Valtrex (viral polymerase inhibitor used for EBV treatment) for a period of 10 days, in a patient with high EBV load exhibiting severe periodontitis (64). EBV genome was quantified by PCR after 1, 5, 8, and 12 weeks post treatment. Interestingly, the viral load was reduced from 0.3 to 0.9 million copies per infected site before treatment to below detection even after 1 week of therapy and remained undetectable 1 year later. This case suggests that herpesvirus screening and subsequent antiviral therapy in conjunction with conventional periodontal therapy may prove more effective in patients with extremely high viral titers. Further research is needed to identify how herpesviruses play a role in pathosis and possible superinfection with bacteria.

Based on the viral genome detection in different oral inflammatory diseases, the presence of EBV, HSV-1, HCMV, HHV-6B, and KSHV is shown to correlate with the pathogenesis of one or more of these viruses. In the subsequent sections, we will therefore focus on these five different viruses representing all the three subfamilies of *Herpesviridae*.

## Clinical manifestations of oral disease associated herpesviruses

### HSV-1 (alphaherpesvirus)

Despite belonging to the same family, members of Herpesviridae can cause vastly different disease processes. HSV-1, a member of the alpha- subfamily, is most commonly spread through direct contact with infected saliva or active peri-oral lesions (65). After primary infection, which often occurs asymptomatically, the virus remains latent in neurons and associated ganglia. The most common site of latency is the trigeminal ganglion. Despite being in the latent life-cycle phase, the virus can infect other individuals via asymptomatic shedding. Reactivation of the virus may be promoted by physical or emotional stress, at which time replication occurs in epithelial cells (66). As a result, mucocutaenous lesions arise. HSV-1 is carried in highest frequencies in developing countries. Worldwide, the prevalence is approximately 90% and approximately 65% in the United States (67).

Primary herpetic gingivostomatitis (PHGS) is a symptomatic primary infection, most commonly occuring early in childhood. PHGS is associated with sore oral lesions, most commonly seen on the tongue, lips, gingival tissues, buccal mucosa and the hard and soft palates (68). The oral lesions may be preceeded 1–3 days by non-specfic symptoms such as myalgia or malaise (69). The severity of symptoms associated with PHGS is thought to be related to the host response, and the amount of time the virus takes to establish latency (70).

Reactivation of HSV-1 may be spontaneous or triggered. Triggers may be physical in nature, such as ultraviolet light, or emotional in nature (70). The most common type of recurrent herpes simplex results is herpes labialis, an extraoral lesion observed on the vermillion border of the lips. Prior to the development of macules, some individuals experience prodromal symptoms. These symptoms include altered sensation, such as burning, itching or tenderness at the site of reactivation. After macules appear, they quickly become vesicular, eventually forming pustular scabs and ulcers. In the vesicular stage they are particularly infective (71). Recurrent herpes simplex, though less common than herpes labialis, is a reactivation that occurs within the mouth. It is most commonly seen on keratinized surfaces, and in immunocompromised individuals. Lastly, herpetic whitlow, presents with vesicles occuring on one or more fingers, likely due to autoinoculation (70).

### HCMV (betaherpesvirus)

HCMV, otherwise known as HHV-5, similarly causes widspread, life-long infection. With a prevalence ranging from 50 to 90%, it is also more common in developing populations (71). Primary HCMV infection is transmitted between individuals via direct contact with bodily fluids. It can be transmitted from mother to baby vertically across the placenta, causing congenital infection, or may infect newborns during delivery or breastfeeding (72). Once infected, HCMV remains with an individual via latent residence in various cell types, including lymphocytes and members of the myleoid lineage (73). While a vast majority of HCMV infections are asymptomatic, reactivation in immunocompromised or immunosuppressed patients, such as those suffering from the acquired immune deficiency syndrome (AIDS) or transplant patients, can cause serious clinical disease (73, 74). Clinical features of HCMV reactivation can range in nature and severity from fever to life-threatening organ failure. Significant sequelae of HCMV in AIDS patients include chronic oral mucosal ulcerations, colitis, and chorioretinitis, which can result in blindness.

HCMV is frequently detected in the oral cavity of immunocompromised (acquired or iatrogenic) individuals where infection appears as a common ulcer, without clinical pathognomonic signs (75). The oral location can be the hard palate, soft palate, tongue, and floor of the mouth (76). Indeed, a significant number of patients with periodontitis are positive for HCMV and virus can be detected in their saliva (77).

### HHV-6B (betaherpesvirus)

Genetically, HHV-6 is related to HCMV, exhibites a wide range of cellular tropism, particularly CD4+ lymphocytes, and induces life-long latent infections (78, 79). HHV-6 infection is widespread and is typically acquired before the age of 2 as protective maternal antibodies are lost (80), although primary infection can occcur in adults (81). In infants, primary infection typically results in self-limited fever and skin rash (*exanthem subitum*, also called roseola) in 10–24% of affected individuals (82, 83). Of note, HHV6 encodes U83B, a chemokine monospecific for monocytic CCR2 which is distinct from HHV6A-derived U83A which activates CCR1, CCR4, CCR5, CCR6, and CCR8 on immune effector cells and dendritic cells. It has been suggested that these differences may result in altered immune cell-subset recruitment for latent/lytic replication (83). In the oral cavity, HHV-6 can be identified in adenoids and tonsils, especially in relation to upper airway infections in children (84). Saliva is the primary mode of transmission and multiple reports have identified HHV-6B DNA in oral carcinoma lesions (85) and endodontic lesions (86, 87). In particular, HHV-6B was significantly associated with large-sized and symptomatic apical periodontitis lesions (87).

### KSHV (gammaherpesvirus)

Human herpesvirus 8 (HHV-8) is involved in the pathogenesis of Kaposi's sarcoma (KS) and was thus termed Kaposi's sarcoma herpesvirus (KSHV). In healthy individuals, primary infection is usually asymptomatic, with sexual contact being the most common mode of transmission. The virus has been detected in saliva, suggesting an alternative mode of transmission. Associated symptoms such as transient fever, lymphadenopathy, and joint pain have been reported. Circulating B-lymphocytes are the major cell of latency (88). HHV-8 has also been associated with a small variety of lymphomas and Castleman's disease.

Kaposi's Sarcoma (KS) is a multifocal neoplasm of vascular endothelial cell origin that was described initially in patients over the age of 60. Since the beginning of the AIDS epidemic, most cases of KS in the United States are associated with HIV infection. About 15–20% of patients with AIDS demonstrate KS (88, 89). HHV-8 is noted within the tumor and is believed to be responsible for development of the neoplasm. HHV-8 has been found in saliva, serum, plasma, throat swabs, and bronchoalveolar lavage fluids and has also been detected in oral epithelial cells and in the oropharynx. This suggests that the oral cavity represents the predominant reservoir of infectious virus. KS manifests as multiple lesions of the skin and oral mucosa, although occasionally a solitary lesion is first identified. The trunk, arms, head, and neck are the most commonly affected sites. About 70% of individuals with HIV-related KS of skin or viscera demonstrated oral lesions. Although any mucosal site may be involved, the hard palate, gingiva, and tongue are most frequently involved. When in the palate or gingiva, the neoplasm can invade bone and create tooth mobility. The lesions begin as brown or reddish macular lesions that do not blanch with pressure. With time, the macules develop into plaques. Pain, bleeding, and necrosis may be a problem. After a biopsy confirming the diagnosis, treatment depends on disease status and symptoms. The treatment is typically highly active antiretroviral therapy (HAART), which has shown a 30–50% reduction in the prevalence of KS. Oral lesions are frequently the cause of major morbidity because of pain, bleeding, and functional interferences. The lesions may be removed surgically or with cryotherapy. Intralesional injection of oral lesions with a chemotherapeutic agent is also an effective option (90, 91). Additional details on herpesvirus infection, miRNA, and oral inflammatory conditions are discussed below.

### EBV (gammaherpesvirus)

EBV is another ubiquitous virus implicated in a wide range of mucocutaneous and systemic diseases. Although EBV can be transmitted by sexual contact, it is primarily spread through infected saliva, resulting in lifelong infection where it preferentially infects and establishes latency in B lymphocytes (92). Indeed, EBV is the causative agent of infectious mononucleosis commonly known as the “kissing disease.” Control of the virus and EBV-associated diseases is efficient in most individuals whereas in immunocompromised individuals, EBV can trigger significant lymphoproliferative diseases, including aggressive malignancies such as Burkitt's Lymphoma. Further, in these patients, EBV is linked to oral hairy leukoplakia which appears as vertical white folds on the lateral and dorsolateral parts of the tongue that do not scrape off although EBV-related tumors of the oral cavity have been described in immunocompetent individuals (93).

In regards to oral inflammatory diseases, two recent meta-analyses based on case-control studies reported that the high frequency of EBV detection in periodontal pockets correlated with an increased overall risk of periodontal disease [Odds Ratio = 5.74–6.20, 95% Confidence Interval = 1.14–12.319, *p* < 0.001; (94, 62)]. Considering ethnicity as a factor, high EBV-detecting frequencies correlated with increased risks of periodontitis in Asians, Europeans, and Americans (*p* < 0.001). Of note, it was reported that periodontal therapy led to decrease in the amount of detectable EBV as well as HSV-1 and CMV in chronic periodontitis patients (*p* < 0.05) compared to baseline (95). Regarding endodontic lesions, a recent review by Hernández-Vigueras and colleagues reported that EBV was identified in a significant number of endodontic diseases (96). In total, EBV was detected in 41% of clinical samples compared to only 2% for controls. In symptomatic periapical lesions, EBV was significant correlated with increased levels of TNF, γ-IFN, IL-1, and IL-12 mRNA (97).

## MicroRNAs: critical players in oral inflammatory diseases

As mentioned above, miRNAs by virtue of simultaneously regulating hundreds of cellular genes can modulate critical biological functions that determine cell fate. Besides cellular targets, miRNAs have been shown to bind and regulate viral transcripts in various infection models and thus act as cellular viral restriction factors. In some cases, the virus can use host miRNAs to regulate their own life cycle. For instance, liver specific miR-122 binds to HCV 5′ non-coding region of the viral genome and facilitates viral replication, but not translation or RNA stability (98). Thus, miRNAs can influence the fate of host-pathogen interactions. These regulatory RNAs fine tune the transcriptome to maintain proper physiological functions. However, under the influence of various stimuli, expression of miRNAs may be altered leading to dysregulated functions. Thus, miRNA can significantly affect the outcome of disease development and progression. Evidently, miRNA levels are modulated during infection and disease states including cancer, autoimmunity, atherosclerosis, Alzheimer's, etc., (99–104). Due to their size, sequence specificity and delivery, miRNA can serve as promising diagnostic markers and therapeutic targets. Although miRNAs have been widely studied in other diseases, identification and characterization in oral diseases have recently begun. In the following section, we will discuss miRNAs altered during oral infections.

### Altered cellular miRNAs in oral inflammatory diseases

There is very limited data on the role and prevalence of miRNAs in the oral cavity. Expression of miRNAs in the head and neck region has been studied mostly in head and neck squamous cell carcinoma. A study evaluating the role of miRNAs in head and neck carcinoma reported that miRNA could function as tumor suppressors or oncogenes (105). In another study, the expression profiles of circulating miRNAs in the serum of patients with high-risk oral lesions (oral cancer, carcinoma *in situ*) were evaluated to identify possible oral cancer biomarkers. Profiles were taken from those with cancer and those who require surgical treatment with intent-to-cure surgical treatment. They found 15 miRNAs that were significantly upregulated and 5 that were significantly downregulated in the presence of disease. Five of these miRNAs showed promise as possible noninvasive biomarkers for detection of oral cancer or high-grade lesions (106).

There are few reports evaluating the differential expression of miRNA in human dental pulps and peri-apical lesions. Recently, we evaluated miRNA expression in healthy and inflamed human dental pulps where we detected expression of 335 miRNAs in these tissues. There was differential expression of miRNAs between healthy and diseased human dental pulps (107). Compared to healthy controls, three upregulated and thirty-three downregulated miRNAs were identified in diseased pulp. In a second study, our group profiled the miRnome in periapical periodontitis, periradicular tissues that are affected by infection of the root canal system (108). Both pulpitis and periapical periodontitis are directly linked to the pathogenicity of the oral microflora. Of the 341 miRNAs detected in periapical tissues, twenty-four showed significant downregulation in diseased samples. In both studies, miRNAs of the miR-181 family were consistently downregulated. MiR-181 is a known anti-inflammatory miRNA and is widely expressed in various cell types with inflammatory potential including fibroblasts, endothelial cells, myeloid cells, lymphocytes, etc., (109, 110). To elucidate the significance of this observation, the impact of miR-181 was assessed using an *in-vitro* infection model using the periodontopathogen derived immunogen, lipopolysaccharide (LPS). Periodontal ligament (PDL) fibroblasts challenged with *Porphyromonas gingivalis* derived LPS exhibit downregulation of miR-181a and miR-181b. This corroborated with the secretion of the key proinflammatory cytokine, IL-8. Interestingly, 3′UTR of IL-8 harbors a functional complementary site for both miR-181a and miR-181b as demonstrated by reporter assays (111). Together, these studies highlights a connection between miRNA, oral microbes in the pathosis of oral inflammatory diseases.

Currently, clinical studies evaluating miRNA expression in relation to periodontitis have shown a possible association of miRNA in disease pathogenesis. MiRNA profiles in healthy and inflamed gingival tissues has been reported in a few studies. MiRNA profiles of 198 gingival tissue samples derived from 86-well-phenotyped subjects with and without periodontitis were evaluated using microarrays. They found 159 miRNAs with significant, differentially expressed profiles between healthy and diseased gingival tissues. Of these, 91 were upregulated and 68 were downregulated in diseased vs. healthy samples. Overall, this study found that specific miRNAs that are overexpressed in healthy or periodontitis-affected gingiva have validated targets for particular genes that were involved in tissue homeostasis and inflammatory/immune responses (112). A study from our lab evaluated miRNA expression in the context of obesity by comparing gingival biopsies obtained with patients with or without periodontal disease. The expression of specific miRNA species in obesity was reported, which could target genes that comprise cytokines, chemokines, collagens, and regulators of glucose and lipid metabolism (113). A similar study was preformed comparing the miRNA profiles of periodontally healthy and diseased human gingival tissues. This study identified 91 upregulated and 34 downregulated miRNAs with at least a two-fold difference in expression between inflamed gingival tissues compared to healthy tissue. Of interest, the miRNAs studied were related to regulation of Toll-like receptors (TLRs). The miRNAs most highly expressed included hsa-miR-126, hsa-miR-20a, hsa-miR-142-3p, hsa-miR-19a, hsa-let-7f, hsa-miR-203, hsa-miR-17, hsa-miR-223, hsa-miR-146b, hsa-miR-146a, hsa-miR-155, and hsa-miR-205. They concluded that periodontal inflammation might involve miRNA pathways related to inflammation and immunity (114). Lastly, a clinical study evaluated miRNA expression in chronic periodontitis tissues in comparison to healthy control samples. The authors performed microarray analysis and reported upregulation of six miRNAs including hsa-let-7a, hsa-let-7c, hsa-miR-130a, hsa-miR-301a, hsa-miR-520d, and hsa-miR-548a with more than eight-fold difference compared to healthy gingival tissues. Many of these miRNAs were linked to periodontal inflammatory pathways that may play a key roles in chronic periodontitis (115).

### Cellular microRNAs in herpesvirus-host interaction: friend or foe?

Cellular miRNAs are also critical regulator of virus tropism and survival inside host cells. Indeed, in higher animals, miRNA pathway is considered as a potent antiviral mechanism as they lack functional siRNA-mediated viral suppression (116–118). As a counter defense strategy, viruses encode for proteins that have evolved to interfere with host microRNA biogenesis or function. For instance, our group showed that HIV Nef protein interacts with Ago-2, a critical component of mi-RISC, thereby disturbing not only the mi-RISC turnover but also dysregulate miRNA sorting and function (119). Similarly, numerous viral suppressor of RNAi (VSRs) are reported in viruses with diverse range of functions encompassing both miRNAs and siRNA pathways, sequestering of dsRNA, binding mature miRNA/siRNA, impaired processing of miRNA/siRNA, etc. (120) Multiple studies have shown antiviral function of cellular miRNAs. Primate foamy virus-1 (PFV-1) transcripts open reading frame (ORF) 2 harbor host miR-32 binding sites. Through its direct interaction with viral transcripts, miR-32 can limit PFV-1 replication (121).

Viruses have evolved strategies to manipulate cellular miRNAs profiles to establish infection and survive antiviral responses. Not surprisingly, host miRNAs are critical in deciding the fate of herpesviruses. Neurotrophic virus HSV-1 target trigeminal ganglia for latency. To understand the molecular mechanism(s) underlying virus tropism, Hill et al. examined the impact of HSV-1 on the host miRNAs expression in primary neuronal cells and showed significant changes in the expression of large number of miRNAs, including miR-146a (122). Higher levels of miR-146a suppressed its target gene complement factor H (CFH), a key gene involved in first-line of defense, and corroborate with an upregulation of the proinflammatory markers cytosolic phospholipase A2 (cPLA2), cyclooxygenase-2 (COX-2), interleukin-1β (IL-1β). A neuron-specific microRNA, miR-138 binds to and repress the levels of ICP0, a viral transactivator of lytic gene expression (123). Mutation in the miRNA binding region rescued miRNA-mediated repression of ICP0. Consistent with this, WT or mutated viruses infected in Vero cells (non-neuronal; miR-138 poorly expressed) and Neuro-2A (neuronal; miR-138 abundantly expressed) cells exhibit increased and reduced ICP0 expression, respectively. Expression of a VEGF-A-responsive miRNA *viz*., miR-132 is also increased upon HSV-1 infection (124). High levels of miR-132 were noticed in the cornea of mice infected with HSV-1. This induction was shown to be VEGF-A mediated as miR-132 expression in animals treated with antibodies against VEGF-A were significantly low compared to controls. A known target of miR-132 is Ras-GAP, which is negative regulator of angiogenesis. Thus, miR-132-mediated induction of HSV-1 may facilitate ocular neovascularization through Ras-GAP signaling. Thus, suppression of the immune system and concurrent activation of proinflammatory microenvironment can provide a favorable niche for HSV-1 survival and propagation in host cells.

Two separate reports from the Tang laboratory demonstrated that cellular miRNAs can acts as pro- or anti-viral factors. While miR-23a was shown to augment HSV-1 replication and survival, miR-101a blocked viral replication (125, 126). Ru et al. showed that increased levels of miR-23a supports viral replication by targeting interferon regulatory factor 1 (IRF1), a multifunctional protein involved in innate antiviral immunity, inflammation, and pro-apoptotic signaling. IRF1 binds to the genes with interferon response elements which involves several anti-viral genes including IFN I and IFN III cytokines. The authors identified radical S-adenosyl methionine domain containing 2 (RSAD2), a key innate antiviral gene, as one of the IRF1-regulated genes. On the contrary, miR-101 suppress HSV-1 replication by targeting mitochondrial ATP synthase subunit beta (ATP5B) (126). Interestingly, a recent study supported the anti-viral role of miR-101 and showed that this cellular miRNA also interferes with the replication of yet another DNA virus *viz*., Hepatitis B virus (HBV) through targeting of FOXO1 gene which drives HBV transcription by binding to the HBV promoter (127). Together, these evidences show host miRNAs in HSV-1-host interactions, and targeting these miRNAs may provide a valuable therapeutic candidate against viruses.

Host miRNA profiles are dysregulated in HCMV infected cells. Ago-1 and Ago-2 CLIP-seq data revealed multiple human miRNAs as differentially expressed in HCMV infected fibroblasts and these miRNAs were unique to post-infection time points (128). At 24 h post-infection (hpi), 5 miRNAs were upregulated and 2 were downregulated, while the 72 hpi dataset showed upregulation of 8 and downregulation of 7 cellular miRNAs. Interestingly, three upregulated miRNAs (miR-182, miR-96, and miR-183) that were common to both time points are part of miRNA genomic cluster and share the exact same “seed” sequence. These cellular miRNA play a critical role in tumorigenesis and their consistently high levels during HCMV infection may benefit virus persistence and spread. Thus, targeting of host miRNAs by viruses allows successful infection and persistence. For instance, HCMV encoded noncoding RNA, miRDE, is shown to bind miRNAs encoded by miR-17–92 cluster through non-canonical interaction. This enhances the degradation of the host miRNAs (129).

Multiple studies have demonstrated host miRNA regulation of KSHV infection and replication. miR-132 is highly upregulated after KSHV infection of primary human lymphatic endothelial cells (LECs) and has a negative effect on the expression of interferon-stimulated genes, thus facilitating KSHV replication (130). Interestingly, this miRNA was also upregulated by HCMV and HSV-1 suggesting a primarily virus-independent host response. This miRNA binds to and repress transcriptional co-activator EP300, which works together with CREB-binding protein (CBP) to initiate antiviral immunity. Thus, herpesvirus employ a common mechanism whereby cellular miRNA is upregulated to suppress antiviral gene expression. Two more cellular miRNAs *viz*., miR-320d and miR-498 are also shown to regulate KHSV reactivation by targeting RTA, an immediate early (IE) viral protein necessary for latency to lytic switch (131). Yan et al. examined HSV-1-mediated impact on KSHV reactivation, and identified various miRNAs that were differentially expressed in latently KSHV-infected BCBL1 cells that were infected with HSV-1. Both miR-320d and miR-498 were downregulated and predicted to bind RTA at different location. Overexpression of miR-320d and miR-498 inhibits RTA levels, thereby suppressing HSV-1-induced KSHV replication.

Host miRNAs also play a key role in regulating herpesvirus infections and reactivation. While some cellular miRNAs are beneficial for virus, other may restrict virus infection. In a screen to identify Epstein-Barr nuclear antigen 1 (EBNA1)-responsive host miRNAs, let-7 family was identified as negative regulator of EBV reactivation in gastric carcinoma and NPC cells lines (132). EBNA1 overexpression induced let-7 family levels, primarily at the precursor transcript levels, leading to EBV reactivation. Two host miRNAs viz., miR-200b and miR-429 were demonstrated to induce EBV-positive cells into lytic replication by downregulating expression of ZEB1 and ZEB2, leading to production of infectious virus (133). These observations reveal evolution of multiple strategies by viruses targeting host miRNA pathways and strongly indicate that miRNA pathway is critical for virus survival.

### Viral microRNAs: a new component in host-pathogen interaction

MiRNAs have been isolated in many organisms including viruses. In 2003, the first viral miRNAs (v-miRs) were discovered in human B cells that were latently infected with γ-herpesvirus EBV (134). Following this discovery, many other v-miRs from human and animal herpesviruses were identified (135). Today, as noted in the miRBase catalog, 34 different viruses are known to encode miRNAs including seven different human herpesviruses.

The typical herpesvirus genome encodes genes expressed during latent and lytic phases. They are further classified into genes that are (i) immediate-early genes regulate primary infection and reactivation from latency, (ii) early genes, most of which encode proteins required for viral DNA replication, and (iii) late genes that code for structural proteins required for viral morphogenesis (6, 7). It has been shown that herpesviruses express miRNAs during both phases of replication. There are unifying themes in the miRNA regulation of herpesviruses including latent/lytic control, immune evasion, and cell survival and proliferation. Given that v-miRs, in general, do not exhibit sequence conservation indicates that each v-miR is likely evolved to acquire unique functions by targeting specific host pathways. Identification of the complete repertoire of host and viral gene targets of each viral miRNAs can provide a thorough understanding of their role in pathogenesis and possibly facilitate development of strategies to therapeutically utilize v-miRs for disease treatment.

#### Alphaherpesviruses encoded mirnas

Among the alphaherpesviruses, miRNAs have been identified in HSV-1 and HSV-2. Cui et al. first reported miRNAs in HSV-1 (136). To date, 18 precursor miRNA have been identified in both HSV-1 and HSV-1 that eventually generate 27 and 24 mature miRNAs, respectively (miRBase release 21; http://www.mirbase.org/). There are vast similarities, up to 70%, between HSV-1 and HSV-2 genomes, however, only few mature miRNAs and even fewer precursor miRNA sequences were conserved across these two viruses (136, 137). For instance, Cui et al., predicted 24 mature miRNAs of which 8 were conserved across HSV-1 and HSV-2 with regard to sequence and genomic location while, out of 5 predicted miRNA precursors only two showed sequence conservation. This suggests that compared to precursor miRNA, mature HSV1/2 miRNAs sequences are strongly conserved by positive selection pressure during the evolution.

**Figure 2:**
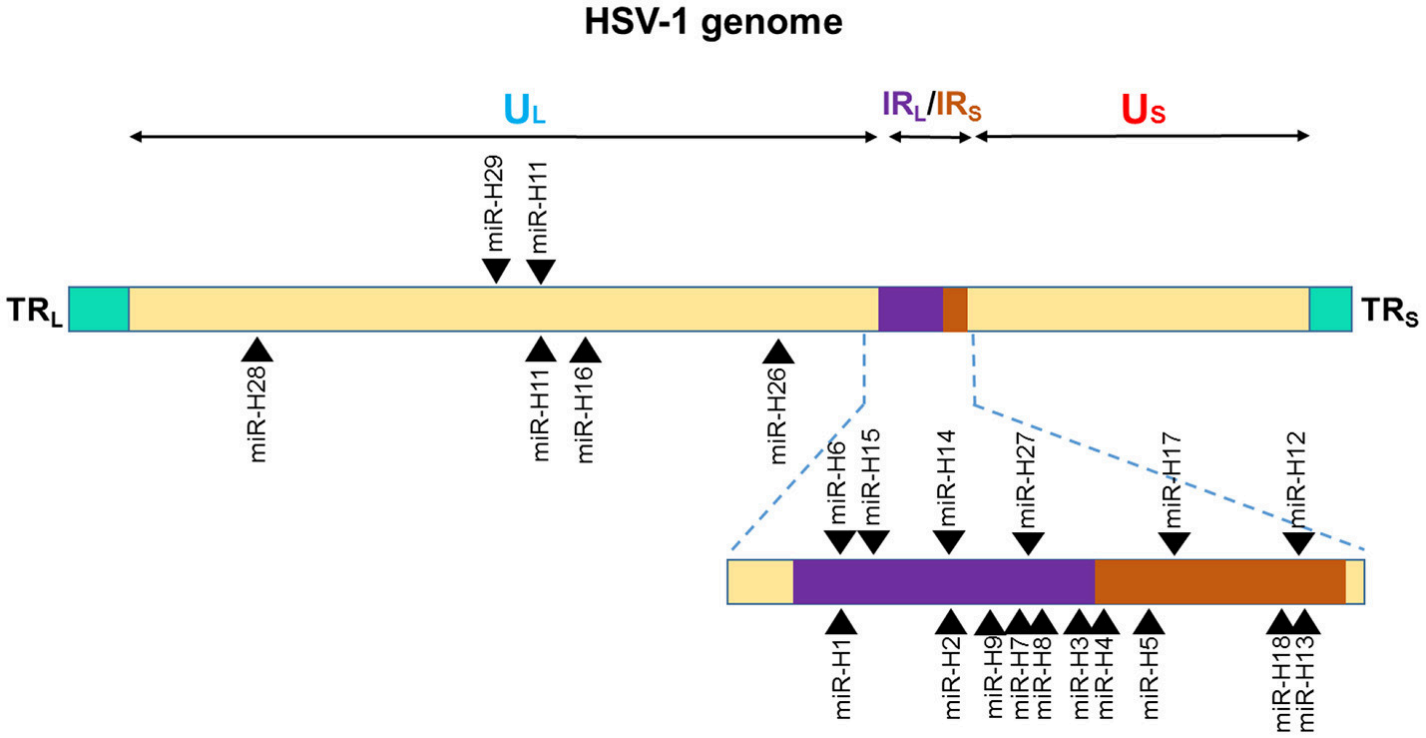
Genomic distribution of HSV-1 miRNAs. The locations of miRNA precursors in the viral genomes are shown as arrowheads. Sense and antisense miRNA precursors are shown above and below the double stranded viral genome, respectively (110, 111, 113). UL and US represent the long and short components of the viral genome. UL is flanked by repeat sequences TRL and IRL while, US is flanked by repeat sequences TRS, and IRS.

Among the 27 mature miRNAs encoded by HSV-1 (miR-H1-8, -H11-18, -H-26-28) more than 50% are in the Latency Associated Transcript (LAT), which generates ~8.3 kb, capped, polyadenylated RNA [(138); Figure 2). All the LAT encoded v-miRs are in the same direction as LAT transcript except miR-H6 (139). The remaining HSV-1 miRNAs are scattered across the viral genome. Interestingly, HSV-2 LAT miRNAs display genomic organization similar to HSV-1, albeit some miRNAs do show virus-specific arrangement. On the other hand, multiple HSV-1 and HSV-2 non-LAT miRNAs show positional difference indicating functional divergence in these miRNAs (138–141). Based on the virus strain, cell type infected, and stage of virus life cycle, different studies have observed variations in the viral miRNA expression strongly suggesting that viral miRNA expression is tightly regulated by the microenvironment. For example, miR-H1 was predicted by Umbach et al. was barely detectable in latently infected neuronal cells; however, the same miRNA is expressed in productive infection and is expressed abundantly in HSV-1-productive samples (139, 140). Similarly, miR-H2 and miR-H4 which are highly expressed in latently infected ganglia could not be detected in primary human fetal foreskin fibroblasts infected with HSV-1 strain 17syn^+^ that was used for establishing quiescent infection (140). Some investigators hypothesize that during productive infection; non LAT-encoded miRNAs are expressed, and result in the suppression of the LAT-encoded miRNA production. Later when HSV enters latent infection, LAT-encoded miRNAs are expressed leading to regulation of other proteins that control latency. The suggestion then, is that these viral.

miRNAs in cooperation with other viral proteins are stage-specific in function to maintain the viral life cycle (141).

Several studies have reported functional roles of HSV-1/2 miRNAs and thus far, evidence supporting their critical role in host-pathogen interaction is well established. By virtue of their antisense to multiple viral open reading frames (ORFs), v-miRs can control viral life cycle at multiple levels (141–145). Various studies showed *cis*-regulation of herpesviral miRNAs. miR-H2-3p is located antisense to an exonic region of ICP0 mRNA and harbors complete sequence complementarity with this key viral gene, a lytic gene activator. Dual luciferase assays showed reduced reporter expression in the presence of miR-H2 (142). Similarly, miR-H3 and miR-H4 are located antisense to the mature ICP34.5 mRNA and overexpression of these miRNAs repress viral transcript and protein expression (143). As mentioned earlier, not only do these v-miRs regulate life cycle switch, they can control virion release. Viral miRNA-mediated regulation of ICP0 and ICP34.5 is also reported in HSV-2 (143, 144). Similar to HSV-1, both HSV-2 ICP0 and ICP34.5 are targeted by identical positional homologs miR-H2 and miR-H3/4 strongly suggesting that viral miRNA regulation of viral transcripts is required for viral maintenance (143, 144). Our study has shown that miR-H1 regulate production and release of virions in oral keratinocytes infected with HSV-1 (strain gL86) consistent with its high levels required during productive infection (146). Indeed, multiple different HSV-1 miRNAs and small RNAs have been demonstrated to modulate virion release suggesting that besides miRNA, viruses may also encode other small RNAs that are required for viral life cycle control (147, 148).

#### Betaherpesviruses encoded mirnas

##### HCMV

**Figure 3:**
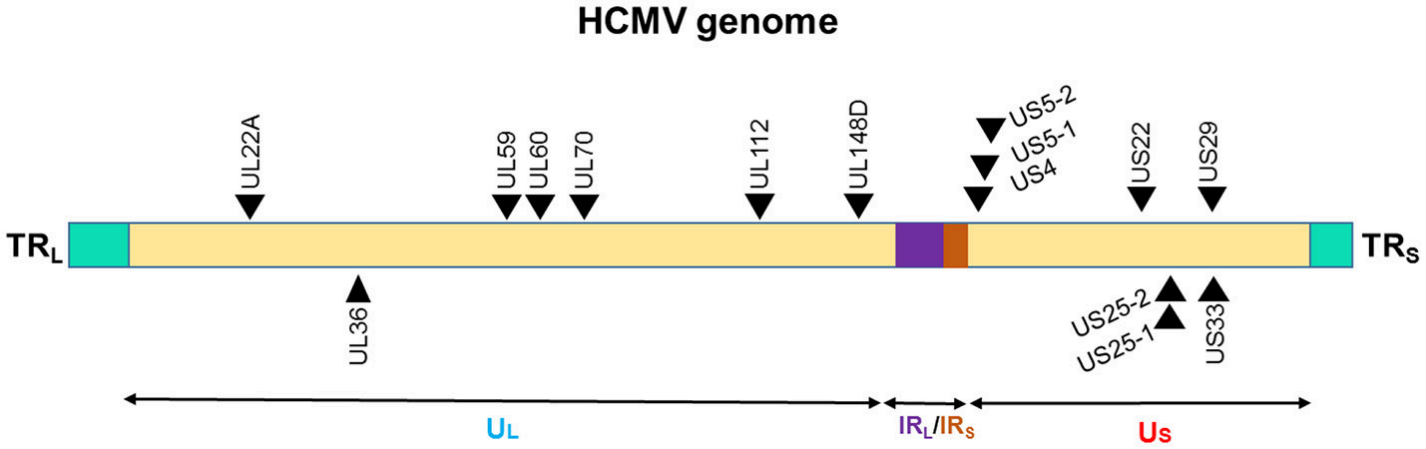
Genomic distribution of the HCMV miRNAs. The locations of miRNA precursors in the viral genomes are shown as arrowheads. Sense and antisense miRNA precursors are shown above and below the double stranded viral genome, respectively (149–151). UL and US represent the long and short components of the viral genome. UL is flanked by repeat sequences TRL and IRL while, US is flanked by repeat sequences TRS, and IRS.

The presence of miRNA genes in betaherpesviruses have been identified in HCMV. Nine miRNA were initially found in HCMV scattered throughout its genome (Figure 3). This number has expanded after cloning and bioinformatics prediction. As of now, 26 mature miRNAs are generated from 15 miRNA precursors. These miRNA genes are produced from several promotor elements (149–151). Initial studies by Grey et al. and Pfeffer et al., identified and validated various set of HCMV miRNAs. In acutely infected fibroblasts, all the tested HCMV miRNAs (US25-1, US25-2, UL22A-1, and UL112-1) exhibit early kinetics expression pattern, with levels continuing to increase over time (150, 151). However, UL70-1 expressed with immediate-early kinetics. Stark et al. using deep-sequencing analysis of infected cells reported similar observation of HCMV miRNAs accumulation with infection duration (128). Indeed, at 72 h post infection (hpi) ~20% of the small RNAs sequenced were HCMV miRNAs. Among the highly expressed HCMV miRNAs were US5-2-3p, UL36-5p, UL22A-3p, US-25-1-5p, UL22A-5p, while levels of US4-3p, UL112-5p, US5-2-5p were among the low expression miRNAs. Only five miRNAs showed drastic change (increase between 24 and 72 hpi) in the expression during time kinetics analysis and included miR-US25-1-5p, miR-US33-5p, miR-US5-2-3p, miR-US22-5p, and miR-US33as-3p. Nonetheless, based on the sequenced reads, HCMV miRNAs primarily grouped as high or low expression miRNAs. It can be noted that HCMV miRNAs are scattered across the genome yet exhibit similar expression profiles suggesting that multiple HCMV miRNAs are required to control viral life cycle and functional modulation of the host. The association of HCMV miRNAs in the Ago-1 or Ago-2 complexes further showed that they are functional and actively recruited into RISC to mediate target gene repression. Both viral and host transcripts are demonstrated as target of HCMV miRNAs (128, 152). Three HCMV miRNAs viz., UL112-1, US5-1, and US5-2 were shown to target multiple genes involves in host secretory pathway thereby interfering with cytokine release and virion formation. Mutant virus lacking these miRNAs show yield reduction and exhibit smaller plaques strongly suggesting that viral miRNAs benefit virus (152). HCMV latency *in vivo* affects multiple tissues including bone marrow, but due to lack of latent tissue culture models, there is limited understanding of miRNA expression in HCMV latency. Its role in viral latency is thus unclear; whereas previously characterized miRNAs are more specific to lytic replication.

##### HHV-6A and HHV-6B

**Figure 4:**
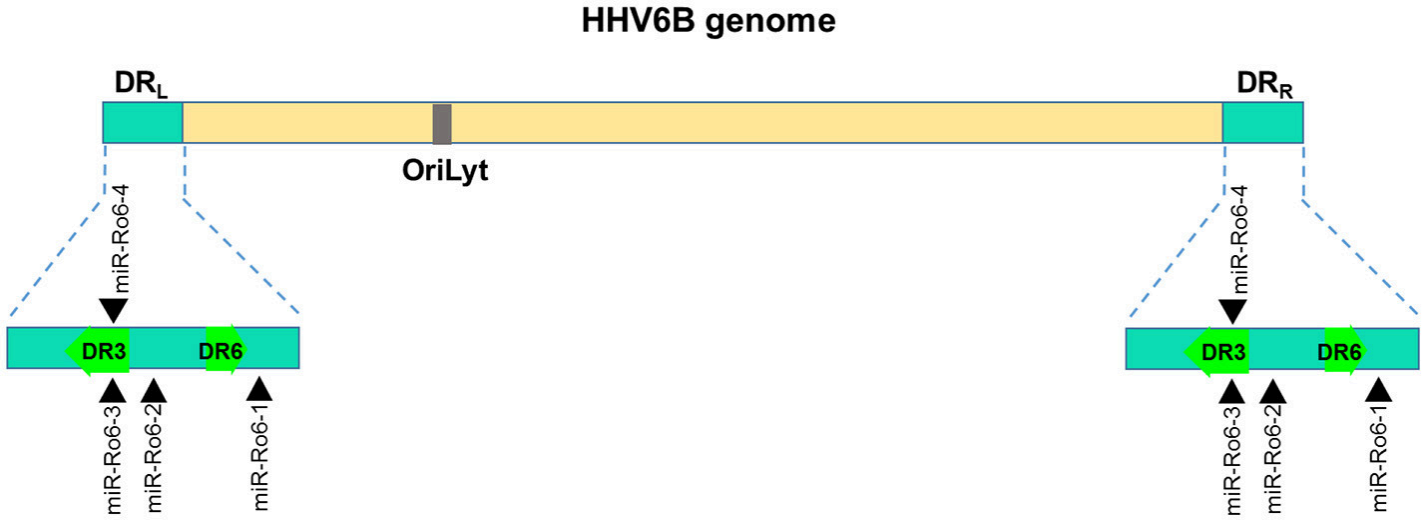
Genomic distribution HHV-6B miRNAs. The locations of miRNA precursors in the viral genomes are shown as arrowheads (153, 154). Sense and antisense miRNA precursors are shown above and below the double stranded viral genome, respectively. Key viral genes (DR3 and DR6) are shown in green color and the orientation is depicted by the arrow direction.

HHV-6A and HHV-6B are highly ubiquitous herpesvirus. They exhibit high sequence homology of up to 90%, but are considered as different viruses. HHV-6B encodes four miRNA precursors that generate 8 mature miRNAs (153). Unlike other herpesviruses where miRNAs are generally expressed as part of latency transcripts, HHV-6B miRNAs identified thus far are restricted within the direct repeats (DR) region of the genome (Figure 4). Although examining the functional consequence of this divergence is not yet studied, it still suggests an evolutionary divergence of HHV-6B encoded miRNAs from other betaherpesviruses. These HHV-6B encoded miRNAs are conserved in HHV-6A signifying their functional importance. Interestingly, even the small repertoire of four miRNAs, one of the HHV-6B encoded miRNA HHV6b-miR-Ro6-2 exhibit strikingly exact seed match with a cellular miR-582-5p, however, a functional significance of this finding remains unanswered (154). Genomic location of HHV-6B miRNAs is antisense to immediate-early viral transcripts suggesting their likely role in regulating viral life cycle.

Employing high throughput sequencing RNA isolated from productively HHV-6A infected cells, Nukui et al., predicted seven HHV-6A encoded miRNA sequences, of which only one *viz*., miR-U86 qualified the miRNA features. This miRNA was shown to target the HHV-6A IE gene U86, thereby regulating lytic replication (154). Targeting IE gens is a common feature of herpesvirus-encoded miRNAs. Unlike, other herpesviruses, HHV-6A mature miRNAs are expressed at very low levels although the precursors are readily detected. Besides, miRNAs, six different viral small RNAs were identified from HHV-6A infected T cells. The authors successfully generated virus from the mutant constructs for all small RNAs and miRNA except sncRNA-U14. This indicates that sncRNA-U14 is critically essential for lytic replication in the T cells. The viral and cellular targets of these HHV-6B miRNA or small RNAs are still largely unknown and future studies may signify their functional role in host-virus interaction.

#### Gammaherpesvirus encoded mirnas

##### KSHV

**Figure 5:**
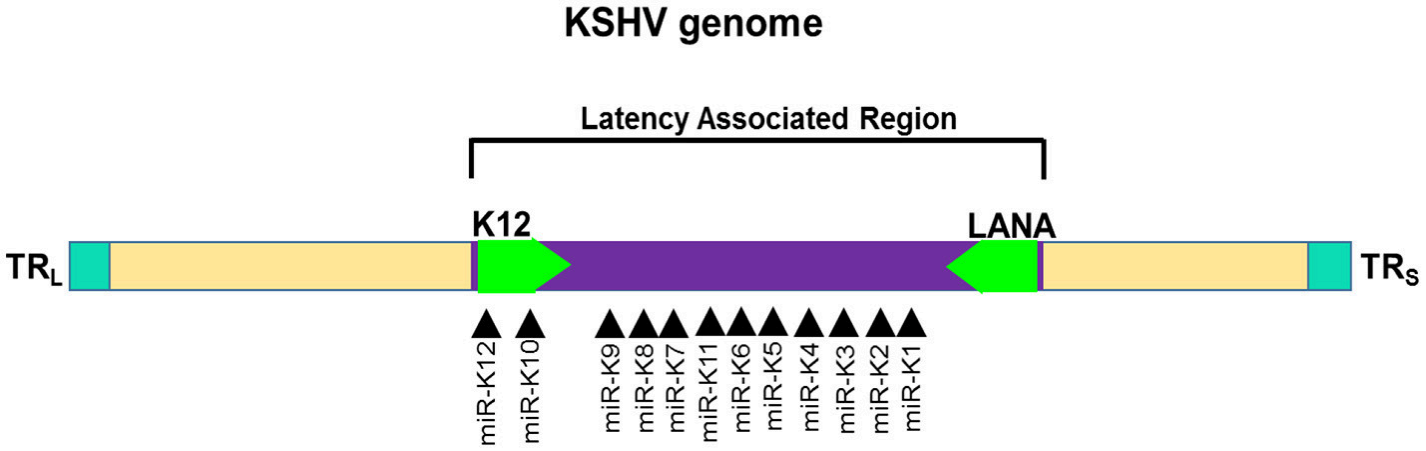
Genomic distribution of KSHV miRNAs. The locations of miRNA precursors in the viral genomes are shown as arrowheads (150, 155). Sense and antisense miRNA precursors are shown above and below the double stranded viral genome, respectively. Key viral genes (K12 and LANA) are shown in green color and the orientation is depicted by the arrow direction.

Among the gammaherpesviruses, miRNAs have also been isolated from KSHV. The miRNAs isolated encode a single cluster of 12 pre-miRNAs with a very high expression in latently KSHV-infected B cells (150, 155). The sequences encoding 10 of the 12 viral pre-miRNAs are located in an intron, while the other two miRNAs, miR-K10, and miR-K12 are located in the viral K12 open reading frame and in the K12 mRNA 3'UTR (Figure 5). All of the 12 KSHV pre-miRNAs are expressed during latency. There is a cluster containing a lytic viral promoter that encodes for the K12 protein, but also miR-K10 and miR-K12 during lytic reactivation. The role of these two miRNAs is not completely understood but seem to play a role during KSHV replication (155).

Several KSHV miRNAs are known to target the same genes or members of the same pathway strongly suggesting that viral miRNAs work in concert. For instance, three KSHV miRNAs viz., miR-K12-1, miR-K12-3, and miR-K12-4-3p bind different sites on the 3′UTR of caspase 3 and suppress expression of apoptosis effector protein (156). Multiple host genes involved in apoptotic pathway are targeted by KSHV miRNAs. Tumor necrosis factor (TNF)-like weak inducer of apoptosis (TWEAK) receptor (TWEAKR) is directly regulated by KSHV miR-K10a leading to increased cell survival upon stimulation with TWEAK. Another pro-apoptotic gene targeted by miR-K10a is Bcl2-associated factor (BCLAF1) (157). Reduced caspase activity in the presence of KSHV miRNAs allows viral persistence in infected cells thereby allowing these oncogenic viruses to contribute to the malignant phenotype. Another mechanism through which KSHV promotes cell survival is by enhancing NFκB activity, a positive regulator of cell survival (158). KSHV miR-K1 targets the 3′ UTR of IκBα RNA, an inhibitor of the NFκB complexes, thereby relieving NFκB to exert its pro-survival activity through induction of several antiapoptotic proteins, including FLIP, Bcl-XL, A1/Bfl-1, cellular inhibitor of apoptosis (c-IAP), etc. (159). Additionally, miR-K1 inhibits viral lytic replication by enhancing NFκB and hence promote viral latency.

An interesting feature of herpesviral miRNAs is sequence homology with host miRNAs. This allows viruses to suppress expression of key cellular genes targeted by host miRNAs. A well-known example is KSHV encoded miR-K12-11 that display complete homology with the “seed” region of miR-155, a critical miRNA with immunosuppressive functions (160). Cellular miRNAs commonly exhibit heterogeneity in the 5′ sequence thereby broadening the target repertoire of miRNAs. These miRs with single nucleotide shift, generated during miRNA processing, in the 5′ seed region are called 5′-isomirs. Similar generation of 5′ isomirs is found in KSHV where heterogeneity in the 5′ sequence enhances homology with the cellular miRNA (161). For instance, the seed region of miR-K10a isomir (miR-K10a+1) exhibits exact sequence homology with miR-142-3p isomer (miR-142-3p-1) thereby allowing viral miRNA to extent the range of target genes including a large set of cellular miRNA regulated genes (162).

##### EBV

**Figure 6:**
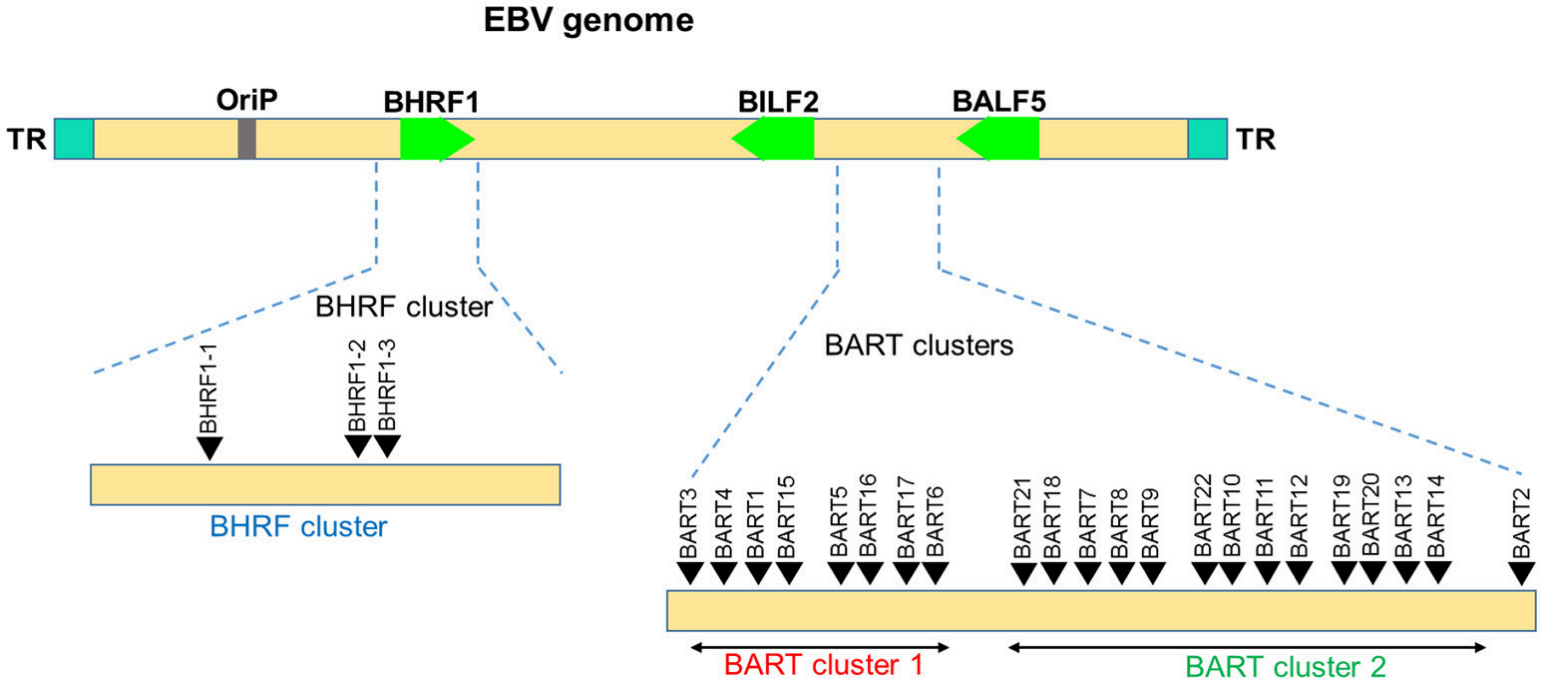
Genomic distribution of the EBV miRNAs. The locations of miRNA precursors in the viral genomes are shown as arrowheads (134, 150, 163). Sense and antisense miRNA precursors are shown above and below the double stranded viral genome, respectively. Key viral genes (BHRF1, BILF2, and BALF5) are shown in green color and the orientation is depicted by the arrow direction.

EBV miRNAs were the first virus encoded miRNAs discovered by Pfeffer et al. (134). Among the human infecting herpesviruses, EBV encodes the most number of miRNAs. This virus is known to encode 25 precursor miRNAs that gives rise to 44 mature miRNAs [135, 151, 164; miRBase version 21]. All the miRNAs are encoded by two major transcripts namely BHRF and BART (Figure 6). Four mature miRNAs are generated from BHRF transcript. BART miRNAs are located in two distinct clusters *viz*., cluster 1 and cluster 2. BART Cluster 1 encoded eight miRNA precursors (BART1, BART3-6, BART15-17), while the larger BART cluster 2 generate 12 different miRNA precursors (BART7-14, BART18-22). BART2 is the only miRNA that is isolated from the rest of BART miRNAs. Although encoded in clusters, expression of EBV miRNAs is not strictly co-regulated and varies throughout the different stages of infection indicating stage-specific requirement of these miRNAs (134, 150, 163). EBV encoded miRNAs have been extensively studied and their roles in cell reprogramming, apoptosis, immune subversion, viral persistence, and latency regulation are well recognized.

Viral miRNAs play a critical role in herpesvirus immune evasion (11, 116). NK cells or CD8+ T cells are involved in the clearance of virus-infected cells. To survive lifelong inside host, herpesvirus have evolved ingenious mechanisms to evade constant threat of host immune cells. miR-BART2-5p mediated suppression of MHC class I-related chain B (MICB), a critical molecule that recognize virus infected cells, protects EBV-infected cells from attack by NK cells and T cells (164). Targeting of host immune activation molecules by multiple EBV miRNAs can be harnessed as potential alternative gene therapy approach to enhance the successful outcome of the grafted cells/organs.

EBV miRNAs, like KSHV, contribute to tumorigenic activity, primarily by suppressing pro-apoptotic pathway. This allow EBV to maintain latency and survival of virus infected cells. The biological significance of viral miRNAs in EBV-associated cancers and clinical manifestation in immunocompromised diseases. EBV miRNAs have been demonstrated to potentiate cellular transformation. EBV can latently infected B cells or transform them to indefinitely proliferating lymphoblastoid cell lines (LCL). Three clustered EBV miRNAs viz., miR-BHRF-1-1, miR-BHRF-1-2 and miR-BHRF-1-3 were shown to promote cell growth and colony formation of LCL (165). Thus, viral miRNAs provide novel therapeutic targets to treat EBV-associated cancers. Viral miRNA repertoire of EBV has been investigated in cancers including nasopharyngeal carcinoma (166), gastric carcinoma (167), and shaping of tumor microenvironment (168) and significant changes in miRNA levels has been reported suggesting significant oncogenic potential.

### Viral miRNAs in oral inflammatory disease

Higher prevalence of herpesviruses in the diseased oral tissues strongly supports their role in augmenting disease. However, although proposed, there is little information regarding the mechanisms as to how they contribute to pathogenesis. The detection of a viral genome only provides information regarding the presence of a virus but no further functional correlation can be derived. Monitoring changes in the levels of bioactive molecules viz., proteins and RNA, can provide novel information on the contribution of viruses to the pathogenesis of oral diseases. Our group for the first time investigated the role of viral miRNAs in oral infection and demonstrated that changes in v-miR expression occurs during disease (169). Microarray analysis of 85 different v-miRs (along with cellular human miRs) in healthy and inflamed tooth pulps detected multiple virus-derived miRNAs. Four v-miRs encoded by HSV-1, HCMV, and KSHV showed significantly higher levels in disease compared to healthy tissue. To test this observation, we further examined the expression of these v-miRs in gingival biopsies in subjects with periodontitis. Interestingly, we noticed that three of the four v-miRs exhibit increase in expression in diseased tissues compared to healthy controls (146). These v-miRs also display similar increased expression in obese periodontitis subjects. This data correlates with the previous reports that demonstrates higher prevalence of HHV genome in various oral diseases and suggest that induced levels of herpesvirus encoded v-miRs may play a possible role in the inflammatory response. These results show that HHV v-miRs can be detected in different oral tissues and can be used as markers of active oral infection.

Transcriptome-wide (both mRNA and miRNAs) studies focusing on the functional role of these disease-associated viral miRNAs, signify their impact on host cell transcriptome. Indeed, altered expression of a large number of mRNAs and miRNAs were observed upon overexpression of v-miRs in two key host cells *viz*., oral keratinocytes and myeloid cells (146, 170, 171). Global pathway analysis revealed important gene networks targeted by these v-miRs including endocytosis, cell movement, immune signaling, etc. Attenuating, but not abolishing, immune responses is a feature of herpesviruses that allows them to persist as a life-long infection in a host with functional immunity. Evidently, these viruses exhibit symptomatic infection in immunocompromised individuals reflecting that fact that immune subversion is closely associated clinical manifestation in herpesviruses. Consistent with this, enforced expression of the v-miRs identified in our studies were found to suppress the inflammatory response by key immune cells viz., primary human macrophages and dendritic cells (171). For instance, miR-H1 and miR-K12-3 transfected myeloid cells exhibit attenuated phagocytosis and altered cytokine secretion upon challenge with whole *E. coli* (171).

Higher levels of HCMV viral miRNAs were observed in human subjects with oral lichen planus (OLP), a T cell-mediated autoimmune disease. Ding et al. examined HCMV miRNAs in the plasma of 95 newly diagnosed, untreated OLP patients (172). Initial screeeing of 23 HCMV miRNAs was performed on 21 OLP patients and 18 healthy controls. Surprisingly, all the HCMV miRNAs were detected in healthy and controls subjects. However, expression of only five HCMV miRNAs viz., hcmv-miR-UL112-3p, hcmv-miR-UL22a-5p, hcmv-miR-UL148d, hcmv-miR-UL36-5p, and hcmv-miR-UL59 were significantly higher in OLP patients compared with healthy controls (172). This corroborates with the increased detection of HCMV DNA in the peripheral blood leukocytes in diseased subjects suggesting higher viral titers in the leukocytes may contribute to the elevated HCMV miRs.

Accumulation of viral miRNAs is oral cancers is consistently noticed in multiple studies. EBV-associated nasopharyngeal carcinoma (NPC) is extensively studied oral caner. MiRNA profiling of tissues from subjects with NPC (*n* = 16) and non cancerous (NC; *n* = 20) revealed higher levels of seven EBV miRNAs, among which ebv-miR-BART7-3p was most significantly increased (173). Ectopic expression of ebv-miR-BART7-3p in EBV-negative NPC cells results in greatly increased metastases in mice strongly supporting the role of viral miRNAs in tumor progression. ebv-miR-BART7-3p-mediated downregulation of phosphatase and tensin homolog (PTEN), a critical tumor supporesor gene, leads to the accumulation of Snail and β-catenin This augments epithelial-to-mesenchymal (EMT) transition and progression. Same resarch group also identified higher EBV BART cluster miRNAs in NPC tissue biopsies (174). Besides, previously described ebv-miR-BART7-3p, they showed ebv-miR-BART1 expression strongly correlate with the pathoclinical features. Mechanistically, ebv-miR-BART1 (similar to miR-BART-7-3p) directly downregulate PTEN levels and enchance phosphorylated AKT, FAK, 130Cas, Shc, and ERK1/2. These findings support that multiple herpesviral miRNA synergistically regulate same gene/pathway to mediate pathological outcome. Another independent study also showed higher levels of ebv-miR-BART7 in the plasma of NPC patients (175). Importantly, subjects that were negative for EBV DNA were positive for ebv-miR-BART7 suggesting that cicrulating viral miRNA provide reliable diagnostic marker to differentiate NPC and NC tissues. *In vitro*, cells overexpressing ebv-miR-BART7 were resistant to cisplatin treatment and display enhanced cell proliferation, promoted cell migration and increased invasion. Employing viral miRNAs as novel diagnostic markers in disease progression or using viral miRNA as therapeutic targets in combination with existing treatment modalities could provide potent and reliable approach to contain oral cancers and infammatory diseases.

## Conclusion and future directions

Host-pathogen interactions are highly intricate relationships that involve numerous factors. The oral cavity is a highly dynamic environment with constant exposure to factors (endogenous or exogenous) that can influence oral tissue homeostasis. Identification and characterization of components that contribute, directly or indirectly, to this functional interaction could provide better understanding of how they can affect host-microbiome equilibrium. Herpesviruses are commonly acquired, often asymptomatic viruses with tropism to oral tissues that are associated with oral disease manifestation. Among the host factors, changes in the cellular miRNAs profiles is consistently shown to be altered in oral infections. Detection of herpesvirus encoded miRNAs and changes in their levels in oral disease have added yet another dimension to our understanding of the already complex host-pathogen interactions. These findings indicate that cellular and viral miRNAs may act as critical triggers in oral disease pathogenesis or may act as markers. The repertoire of disease-regulated miRNA may not only provide novel diagnostic targets unique to each disease, they may also serve as promising therapeutic targets. However, larger cohort clinical studies from different geographical locations and similar disease parameters are required to test the therapeutically relevant miRNAs. Global targetome analysis of disease-associated miRNAs can provide a holistic view of their role in disease. Not only this will advance our understanding of the role of viral miRNA in pathogenesis, it will open new avenues of research to employ therapeutic targets. In particular, knowledge on immune evasion strategies of viral miRNAs can be adapted to assess the applicability and possibilities of these viral miRNAs as a tool for use in gene therapy. For instance, host immune response against non-self-antigens leads to graft rejection. Employing immune subversion mechanisms targeted by viral miRNAs may be considered as novel gene therapy approach, however, various issues regarding the off-target effects remains (176). Nonetheless, with the advancement in high throughput technologies in miRNA profiling, targetome analysis, microbiome characterization, and miRNA delivery methods, oral health care providers can envision these tiny, enigmatic regulatory RNAs as a near future diagnostics as well as treatment modality.

## Author contributions

AN and SN conceptualized the review. AN prepared all the figures and compiled the tables. AN, AS, JS, DS, and SN wrote the manuscript.

### Conflict of interest statement

The authors declare that the research was conducted in the absence of any commercial or financial relationships that could be construed as a potential conflict of interest.
